# Analysis of Fatigue and Healing Properties of Conventional Bitumen and Bio-Binder for Road Pavements

**DOI:** 10.3390/ma13020420

**Published:** 2020-01-16

**Authors:** Elena Gaudenzi, Fabrizio Cardone, Xiaohu Lu, Francesco Canestrari

**Affiliations:** 1 Department of Civil and Building Engineering and Architecture, Università Politecnica delle Marche, Via Brecce Bianche, 60131 Ancona, Italy; f.cardone@staff.univpm.it (F.C.); f.canestrari@staff.univpm.it (F.C.); 2 Nynas AB, SE-149 82 Nynäshamn, Sweden; xiaohu.lu@nynas.com

**Keywords:** fatigue, self-healing, bitumen, bio-binder

## Abstract

The analysis of fatigue behavior of bituminous binders is a complex issue due to several time-temperature dependent phenomena which interact simultaneously, such as damage accumulation, viscoelasticity, thixotropy, and healing. The present research involves rheological measurements aimed at evaluating the fatigue behavior and compares the self-healing capability of two plain bitumen and a bio-binder obtained by partially replacing one of the plain bitumen with a renewable bio-oil. Healing potential was assessed by means of an experimental approach previously implemented for modified bitumen and bituminous mastic and based on the use of a dynamic shear rheometer (DSR). The effects of some variables such as bitumen type, bio-oil addition, and aging on the healing potential of binders were taken into account. Results showed that the above-mentioned method for healing analysis is also suitable for conventional and bio-add binders. Outcomes of the experimental investigation highlight that fatigue and self-healing are mainly dependent on binder consistency and also affected by aging. Finally, the addition of bio-oil may induce even better performances in terms of healing potential compared to conventional bitumen, especially in aged condition.

## 1. Introduction

Fatigue cracking is one of the major distresses in asphalt pavements and it consists of the formation and propagation of micro-cracks within the bituminous components until failure. The phenomenon arises due to the decreased relaxation capability of the binder phase as a result of aging, temperature variation, moisture, and repeated cyclic loading. Thus, fatigue needs to be studied as bitumen-associated damage phenomenon [1].

In the last decades, experimental studies revealed the “self-healing capability” of bituminous material, generally regarded as the intrinsic response of bitumen to reduce the generated micro-cracks within the bituminous body [2,3]. 

Therefore, several studies have been focused on finding solutions to improve the self-healing of bitumen, such as the research of the optimal healing temperatures and time required for full healing using an induction heating method [4,5,6], adding extenders (e.g., used cooking oil) to soften aged binder [4,7,8], or the introduction of microcapsules containing rejuvenator in aged bitumen [9,10,11,12]. 

Many factors can affect the self-healing capability of bituminous binders, such as bitumen composition, temperature, loading (i.e., strain amplitude, frequency, and rest-period), and aging conditions. In general, bitumen chemistry plays a fundamental role on adhesive and cohesive self-healing components, which are responsible for flowing and wetting of crack faces as well as diffusion and randomization of the inner structure, respectively. Hence, adhesive and cohesive self-healing components prevent propagation of cracks and allow a significant recovery of mechanical properties during time [13]. 

Temperature also has a strong influence on self-healing capability. At a higher temperature, bitumen is less viscous, consequently being able to repair itself quickly. Of course, each different asphalt binder has different optimal self-healing temperature, depending on both chemical and visco-elastic properties. Also, the healing time represents an important factor. In fact, the self-healing rate of asphalt binder and mixture increases with the duration of the unloading phase (i.e., rest periods) [5]. 

Furthermore, the aging of bitumen during service life affects the behavior of the material; however, results in literature are often controversial. According to some studies, with aging, the binder stiffness increases and its relaxation capacity decreases, resulting in a more brittle material with a reduced healing effectiveness [4,11]. On the contrary, a positive effect was found in terms of healing capability when including a certain amount of aged bitumen within modified bituminous binders [3,13].

In addition to self-healing, other reversible phenomena may occur within the bituminous material under loading conditions which directly affect the evolution of viscoelastic properties (e.g., complex modulus) making it difficult to interpret fatigue testing. These phenomena can be listed as follows: Self-heating (due to viscous dissipation within the material), non-linearity (instantaneous loading level dependence of the viscoelastic properties of the material), and thixotropy (a form of structural non-linearity related to reversible micro-structural change during loading and unloading phases) [14]. Therefore, it is important to distinguish between recovery due to real healing and that due to other intrinsic reversible phenomena of viscoelastic materials in order to perform a reliable analysis of the fatigue damage state, which properly takes into account healing effects. In this regard, the self-healing capability of bituminous materials has to be fully investigated in order to understand how this property can delay or slow down the accumulation of fatigue damage within bituminous materials and systems (e.g., asphalt pavements). To this aim, current interlaboratory research activities coordinated by RILEM-Technical Committee 278-CHA “Crack-Healing of Asphalt Pavement Materials” have been devoted to comparing and validating different testing protocols [13,15].

Nowadays, the need to guarantee high safety and durability levels to road structures without increasing costs and environmental impacts is arising great interest. Several sustainable technical solutions have been adopted in road application so far, such as the use of reclaimed asphalt (RA), warm mix asphalt, as well as cold paving techniques with consequent economic and environmental benefits [16,17]. Nevertheless, these technical solutions do not totally solve the issue around the materials’ renewability. Therefore, one of the main current effort is to move towards “greener” solutions by using renewable or bio-based materials. Despite the chemical and mechanical properties of certain bio-binders having been widely investigated [18,19,20,21], no research works have been focused on the effects of bio-materials on the fatigue and healing properties so far. 

The main objective of this research is to evaluate the effect of bitumen type and a bio-based oil on the self-healing capability, and its impact on the overall fatigue resistance of binders. To this end, an innovative experimental approach previously implemented for modified bitumen and bituminous mastic [3,13] was adopted to assess self-healing potential taking into account, also, the concurrent thixotropic phenomenon. The test method is based on running and analyzing the data collected by dynamic shear rheometer (DSR Anton Paar, Rivoli TO, Italy) fatigue test (i.e., time sweep test) consisting of multiple loading phases alternated with rest periods at a specified damage level. Based on this approach, fatigue resistance and self-healing capability of two plain bitumens and one bio-binder (bitumen added with a bio-oil) were assessed. In addition, each binder was tested both in unaged and aged conditions in order to evaluate the influence of aging on the healing properties.

## 2. Materials 

A plain 50/70 penetration grade bitumen was selected as reference material (coded as B.50/70). The bitumen B.50/70 was also used as a base bitumen for preparing a bio-binder which contained 10% (by weight) of a renewable bio-oil that is a residue generated in the processing of a by-product from wood pulp and paper industry. In particular, it is produced (refined) from crude tall oil (CTO). This binder is coded as B.50/70 + A10. The main characteristics of the bio-oil as well as the details on the preparation of the bio-binder can be found elsewhere [19]. As the bio-oil addition results in a consistency reduction of the base material [19], a softer plain bitumen (coded as B.115) having a penetration grade similar to that of the bio-binder was also considered for comparison purposes. 

All three binder samples were investigated in both unaged and long-term aged conditions. The long-term aging was performed by the Rolling Thin Film Oven Test (Matest S.p.A., Treviolo, Italy) (RTFOT, EN 12607-1) aged samples by means of Pressure Aging Vessel (Prentex Alloy Fabricators Inc, Dallas, TX, USA) (PAV, EN 14769). In total, six samples are included in this study, and their conventional properties are listed in Table 1. 

**Figure 1:**
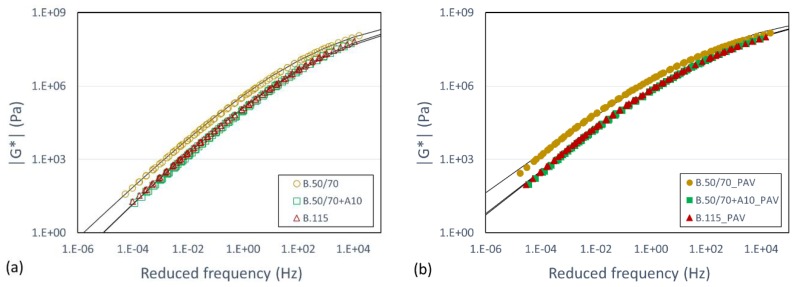
Master curves of investigated bitumen in (**a**) unaged and (**b**) aged conditions.

Furthermore, Figure 1 shows the master curves of the complex shear modulus norm $\left|G^{*}\right|$ at a reference temperature of 30 °C in order to have a comprehensive picture of the rheological response of three investigated binder in both unaged and aged conditions. The experimental data were gathered by performing frequency sweeps at different temperatures by means of DSR. The modified Christensen-Anderson-Marasteanu (CAM) model [1] was adopted to relate $\left|G^{*}\right|$ and the testing frequency, by applying shift factors based on the Williams–Landel–Ferry law [22]. 

The results in Figure 1 show that the bitumen B.50/70 + A10 and B.115, characterized by the same consistency, have a similar complex modulus trend, while bitumen B.50/70 is always stiffer over the entire frequency domain in both aged and unaged conditions. Furthermore, as expected the different rheological response due to the aging (i.e., stiffness increase) is clearly shown in each material. In particular, curves tend to approach to similar values at higher frequencies (or lower temperatures) whereas they deviate more at lower frequencies (or higher temperatures).

## 3. Experimental Program and Test Procedures 

Rheological characterization aimed at evaluating the self-healing potential of all binders in both aging conditions was conducted by means of a DSR in a plate–plate configuration by running cyclic tests in strain-controlled mode. 

Specifically, the healing potential analysis was based on a testing protocol consisting of a time sweep interrupted by multiple rest periods aimed at simulating the sequence of loading and recovery phases which occur in in-service pavements. Indeed, multiple rest periods are expected to guarantee material to properly manifest its self-healing capability [13]. In this study, healing characterization was performed according to the following conditions:

(a) testing in iso-stiffness condition, i.e., each binder is tested at the temperature required to have similar stiffness compared to the other binders;
(b) testing in iso-thermal condition, i.e., each binder is tested at the same temperature regardless of stiffness.

The choice to perform the tests in these different conditions has the objective of identifying which parameter between temperature and stiffness mostly affects the self-healing capability of material. 

Before performing healing tests, all binders were subjected to strain-sweep test in order to identify the limit of the linear viscoelastic (LVE) behavior at different temperatures (5% reduction in G* criterion). LVE limits were assumed as the reference values from which the strain levels in subsequent tests were chosen, with the aim of investigating the material in the non-linear viscoelastic region in which damage likely increases. Strain-sweep tests were performed at five temperature (10, 15, 20, 25, and 30 °C) and 10 Hz.

Each binder was also subjected to isochrone test at different temperatures in order to assess the temperature dependency of stiffness. This enables the evaluation of the iso-stiffness conditions of all binders, i.e., the temperature associated to a given initial stiffness for each material. Isochrone tests were performed at the same frequency selected for healing test (i.e., 10 Hz) and three temperatures by applying a strain level γ = 3%. 

After the preliminary rheological characterization, binders were subjected to fatigue analysis through a time-sweep test with multiple rest-periods, or a healing test, in strain-controlled mode according to the testing protocol developed by Canestrari et al. [13]. In particular, the healing test consists of performing oscillatory tests, alternating loading phases with multiple rest periods. The duration of each rest period was set equal to 30 minutes for the whole test. The protocol provides a minimum of 12 load-rest phases, but in this investigation a reduced number, ranging between five and seven, was applied in all test conditions. During the test a frequency of 10 Hz was adopted, whereas the test temperature was varied depending on the need to have iso-stiffness or iso-thermal conditions. In the former case, for each binder the test temperature was selected from the analysis of isochrone test results to guarantee an initial iso-stiffness level of 3 MPa for all binders. Selection of this iso-stiffness value is to ensure tests at intermediate temperatures (i.e., between 20 and 35°C) which allow the occurrence of the interdiffusion process that is assumed as the main self-healing mechanism [13]. As far as the iso-thermal condition is concerned, a temperature of 25 °C was directly selected as representative of the intermediate temperature regardless of binder stiffness. 

The strain amplitude level was selected to induce significant micro damage within the samples during a reasonable testing time allowing detectable self-healing capability. Thus, for each binder and test conditions the adopted strain level was corresponding to an increase of LVE limit, previously identified with the strain-sweep test, ranging between 15% and 25%. Such a choice also ensured all binders to be investigated at the same damage level beyond the linear viscoelastic domain at the same time minimizing non-linearity effects. Two replicates were tested for each binder and test condition. 

### Self-Healing Capability Analysis

The evolution of the loss modulus $\left|G^{*}\right|\cdot \sin\delta$ during the healing test was selected as a characteristic parameter of the damage accumulation as well as self-healing capability analysis, since it is representative of the change in dissipated energy. Therefore, $\left|G^{*}\right|\cdot \sin\delta$ was continuously monitored during the entire test. 

**Figure 2:**
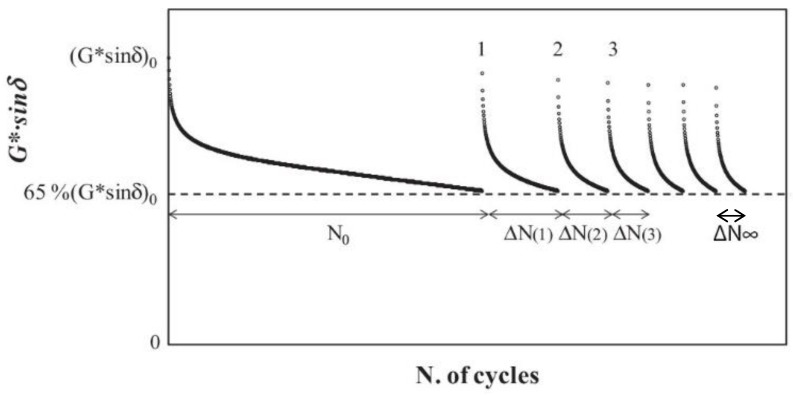
Evolution of $\left|G^{*}\right|\cdot \sin\delta$ versus number of loading cycles: General results.

**Figure 3:**
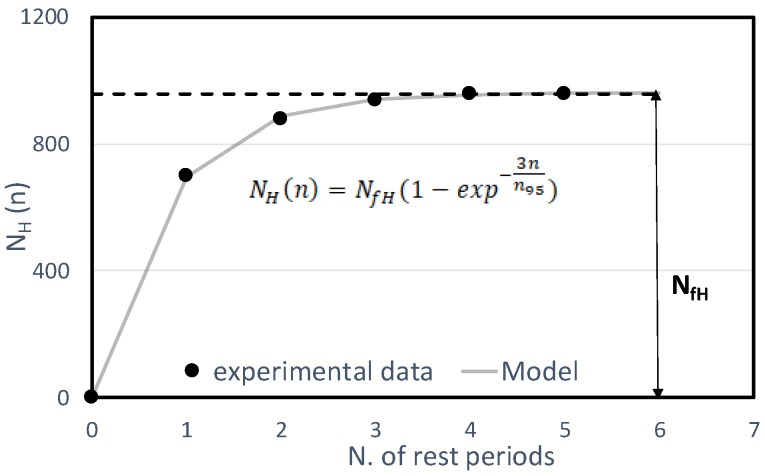
Self-healing contribution versus number of rest periods.

Each loading phase stopped when the $\left|G^{*}\right|\cdot \sin\delta$ dropped down to 65% of its initial value (i.e., 35% loss modulus reduction which represents the selected damage level) and started again after 30 minutes. During each rest period, the stiffness recovery of the material was also monitored by applying a very low strain equal to 0.01% in order to avoid material damage. Typical healing test results are shown in Figure 2 in terms of 35% loss modulus reduction per each loading phase. The main parameters able to quantify the overall fatigue endurance limit of each binder, according to the selected modeling approach [13], can be summarized as follows:

(1) $$N_{\mathrm{Fat}}=N_{0}+N_{\mathrm{fH}}$$

where N_Fat_ is the effective number of loading cycles that the material withstands before failure considering the “n” rest periods, N_0_ is the number of loading cycles in the first loading phase required to reach the prefixed damage level (i.e., 35% reduction of the initial $\left|G^{*}\right|\cdot \sin\delta$ value), and N_fH_ is the cumulative self-healing capability due to the rest periods defined as follows:

(2) $$N_{\mathrm{fH}}=\;\underset{n\to \infty }{\lim}N_{H}\left(n\right)$$

(3) $$N_{H}\left(n\right)=\overset{n}{\underset{ì=1}{\sum }}∆N_{H}\left(i\right)$$

(4) $${\Delta N}_{i}={\Delta N}_{H}\left(i\right)+{\Delta N}_{\infty }$$

where N_H_ is the overall self-healing contribution after “n” rest periods, ∆N*_i_* is the number of loading cycles after each “i-th” rest period, which are necessary to reach the same damage level selected (65%$\left|G^{*}\right|\cdot \sin\delta$), ΔN_H_(i) is the self-healing contribution (variable depending on the “i-th” rest-period considered), and ${\Delta N}_{\infty }$ is the thixotropy contribution (constant for each rest/load phase). In particular, the self-healing contribution ΔN_H_(i) decreases with the amount of rest applied and vanishes after a certain number of rest periods. Thus, after a high number of rest periods (virtually infinite) the cumulative self-healing contribution, defined as the self-healing potential, converges to a finite value N_fH_ as depicted in Figure 3. Finally, the healing index (HI) can be calculated as follows:

(5) $$\mathrm{HI}=\frac{N_{\mathrm{Fat}\;-{\;N}_{0}}}{N_{0}}\;\times \;100=\frac{N_{\mathrm{fH}}}{N_{0}}\;\times \;100$$

Considering that N_0_ represents a sort of resistance to micro-cracking due to fatigue damage (i.e., number of cycles required to reach a given damage level), HI can be taken as a good indicator of material capability to recover part of its viscoelastic properties when rest periods are applied. Thus, the higher HI, the higher the self-healing potential of material.

## 4. Analysis of Experimental Results

### 4.1. Strain-Sweep Test

Table 2 reports strain-sweep test results for the investigated binders in both aging conditions (unaged and aged) at different temperatures. As expected, the LVE limit γ_LVE_ decreases with the decrease in temperature for all tested binders. Moreover, it can be observed that bio-binder and bitumen having the same consistency (i.e., penetration grade) show similar γ_LVE_ values in both unaged and aged conditions, while the harder bitumen B.50/70 shows lower values at all test temperature. 

In addition, the percentage variation of γ_LVE_ between unaged and aged binders Δγ has been calculated at each temperature in order to evaluate the aging effect on the LVE behavior of material (Table 2). 

Results show that Δγ for softer binders seems to be more affected by aging at each temperature investigated compared to the bitumen B.50/70, however the aging effect on Δγ values is less marked as the temperature decreases. Furthermore, no direct effects due to the bio-adding can be seen on γ_LVE_ of bio-binder, suggesting this parameter is rather dependent on the consistency of the materials.

### 4.2. Isochrone Test

**Figure 4:**
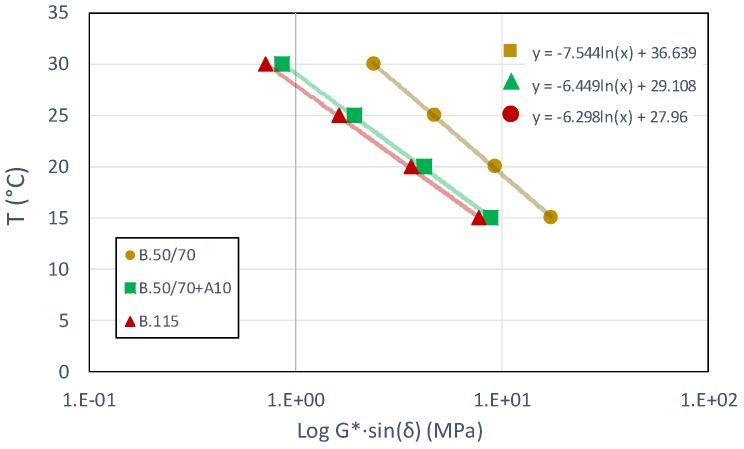
Typical isochrone test results.

Figure 4 shows the typical results of the isochrone tests carried on unaged binder. In particular, values of $\left|G^{*}\right|\cdot \sin\delta$ were plotted in a semi-logarithmic scale as function of test temperature and a linear regression was run to fit experimental data with the aim to identify the temperature dependency of stiffness. Finally, it is possible to back calculate the temperature required to guarantee the desired target stiffness from the linear fitting.

Table 3 summarizes the iso-stiffness temperatures for stiffness values ranging between 1 to 12 MPa for all investigated binders in both aging conditions. 

The temperature values in Table 3 are calculated based on the linear equations, therefore they represent indicative values which can undergo a variation of ±1 °C in order to practically achieve the required stiffness.

As expected, for the binders characterized by higher penetration grade, lower temperatures are needed to reach the same stiffness value. Moreover, since aging causes material stiffening, higher temperatures are required for the aged binder to reach the same $\left|G^{*}\right|\cdot \sin\delta$ value. However, from Table 3, a similar change in temperature to cause a stiffness increase or to have the same stiffness level between unaged and aged binders can be observed regardless of considered material. Moreover, looking at the results in Table 3 related to the B.50/70 + A10 and B.115 binders having the same grade, the temperature values are very similar whatever the stiffness and aging condition. This finding shows that the bio-based oil does not affect the thermal sensitivity as well as the aging effects experienced by the investigated binders. 

### 4.3. Healing Test

Based on the results from strain-sweep and isochrone tests, Table 4 sums up the testing parameters selected for the healing test to obtain the iso-stiffness and iso-thermal conditions. 

As bitumen rheological behavior is strongly dependent on strain level applied, it was chosen to impose a strain level γ_heal_ just above the LVE region. In particular, all materials were investigated in the non-linear viscoelastic domain by considering an increase of 15–25% with respect to the LVE limit. 

**Figure 5:**
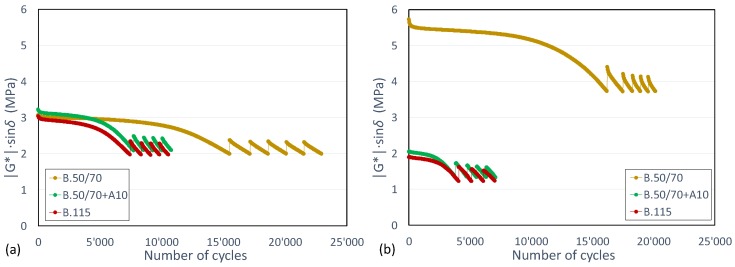
Comparison among unaged binders in (**a**) iso-stiffness and (**b**) iso-thermal conditions in terms of evolution of $\left|G^{*}\right|\cdot \sin\delta$ versus number of loading cycles.

Figure 5 shows typical results of the healing test performed on the three unaged binders in iso-stiffness (initial loss modulus equal to 3 MPa) and iso-thermal (T = 25 °C) conditions. Specifically, for each binder the $\left|G^{*}\right|\cdot \sin\delta$ reduction due to the loading is plotted as a function of the number of loading cycles.

For all binders, the decay curves gathered in the first loading cycles show a typical fatigue test trend for which, after an initial rapid decrease in modulus, a quasi-linear evolution and a subsequent abrupt loss in mechanical properties can be observed. After the first loading phase, the recovery of the modulus due to the rest slightly reduces and the modulus reduction during the subsequent loading phases gets faster. Contrary to what is recommended in the testing protocol [13], which was implemented for modified bitumen suggesting a minimum of 12 load-rest phases, it can be observed that for both unmodified bitumens and the bio-binder the repetition of fewer load-rest cycles (usually ranging between five and six) was suitable to achieve a decrease rate of modulus that no longer significantly changes. 

From the analysis of both plots, it can be seen that the damage accumulation is similar between bio-binder and bitumen B.115 and it is faster than in the binder characterized by a lower penetration grade, regardless of the applied rest periods. This finding is consistent with previous studies confirming that for a not-significant level of strain, harder bitumens have a better fatigue response than softer ones [23].

**Figure 6:**
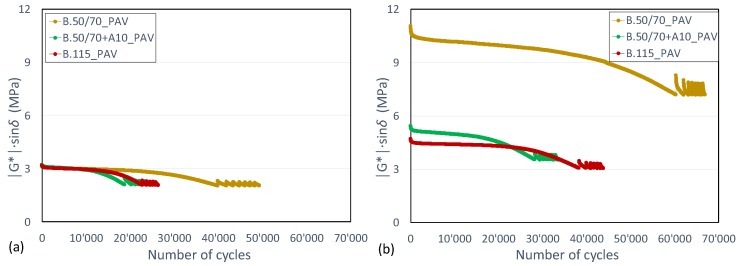
Comparison among aged binders in (**a**) iso-stiffness and (**b**) iso-thermal condition in terms of evolution of $\left|G^{*}\right|\cdot \sin\delta$ versus number of loading cycles.

Figure 6 shows that similar trends were obtained for aged binders as well. This result is consistent with those reported in Figure 5, highlighting that a binder characterized by a higher consistency shows enhanced fatigue behavior compared to a softer binder.

Experimental data from each healing test were then analyzed according to the modeling previously introduced in order to determine the main parameters describing fatigue, healing, and thixotropy properties. Average results are listed in Table 5 and Table 6.

As for N_fat_ parameter, representing the effective number of loading cycles material withstands before failure considering the rest periods, its values confirmed what previously stated based on raw test data (Figure 5 and Figure 6). Similar trend was noted for N_0._ Specifically, the binders characterized by a higher penetration grade showed comparable N_0_ values and lower than the harder binder in both aging conditions. Moreover, whatever the binders, N_0_ increases with aging, and it is probably due to the not-significant strain applied. This would confirm for some materials (e.g., depending on composition and consistency) that aging effects on the fatigue response are dependent on the strain level [24,25]. Similarly, based on the strain level applied, higher N_0_ are observed for binders characterized by a higher stiffness.

Table 5 and Table 6 also report the values of parameter n_95_ that is the number of rest periods necessary to reach the 95% of the asymptotical value N_fH_ (Figure 3), in other words after this number of the rest period the material has almost achieved its complete healing potential. The values of n_95_ seem to be mainly affected by the aging conditions rather than by the binder type. In particular, the n_95_ values are higher for the aged binders, denoting that the aged binders are more prone to preserve the self-healing capability for a higher number of rest period. 

**Figure 7:**
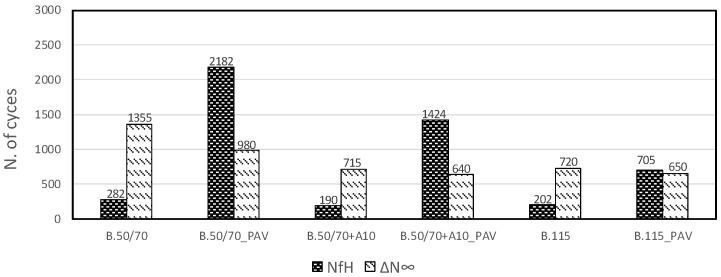
Model parameters for all investigated binders in iso-stiffness condition.

**Figure 8:**
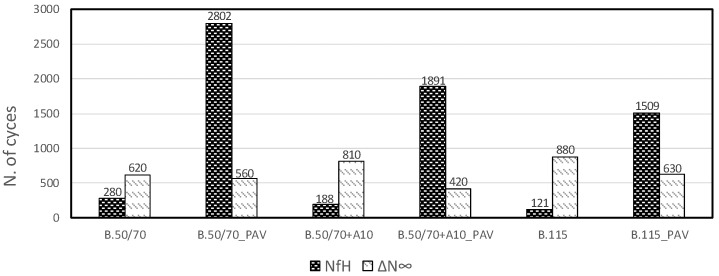
Model parameters for all investigated binders in iso-thermal condition.

Figure 7 and Figure 8 show the results for all binders in terms of cumulative self-healing capability N_fH_ and thixotropy component ΔN_∞_ for iso-stiffness and iso-thermal conditions, respectively.

From the iso-stiffness analysis of Figure 7, it can be seen that N_fH_ values for unaged binders are rather low, whereas the aged conditions show higher values of N_fH_ for all investigated binders. Moreover, aged binders characterized by a higher penetration grade show lower self-healing capability parameter. However, the aged bio-binder seems to guarantee an enhanced healing aptitude compared to bitumen B.115 despite similar consistency. In regards to the parameter related to the thixotropic behavior, the higher the ΔN_∞_ value, the higher the thixotropic effect experienced by the binder. Figure 7 shows that in iso-stiffness conditions ΔN_∞_ parameter is higher for the harder bitumen compared to the softer ones, which show similar values. Moreover, thixotropy component decreases with the aging especially for the harder bitumen.

Iso-thermal results of Figure 8 confirm for N_fH_ parameter the trend obtained in iso-stiffness conditions, highlighting better healing potential of bio-binder compared to the one having the same consistency.

As regards the thixotropy contribution in isothermal conditions, the aging effects are evident especially for the softer binders, resulting in a decrease in thixotropy. Moreover, the comparison between iso-stiffness and iso-thermal conditions shows that ΔN_∞_ parameter is mostly dependent from temperature [26].

From the comparison between Figure 7 and Figure 8 it can be seen that the N_fH_ parameter for unaged binders does not seem to be affected by temperature or stiffness, in fact all materials show similar values both in iso-thermal and iso-stiffness conditions. On the contrary, the healing aptitude of aged binders seems to be dependent on the temperature, in particular lower temperatures result in higher N_fH_. It is important to highlight that this finding can be assumed valid in the range of temperature investigated.

**Figure 9:**
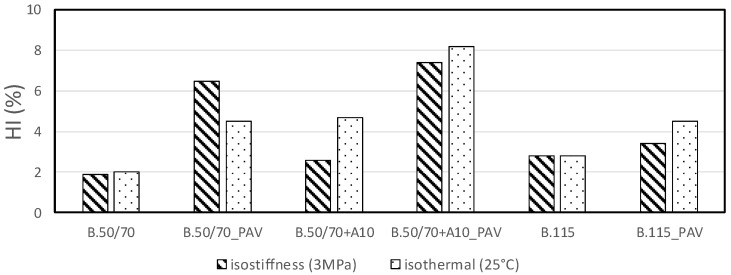
Healing index (HI) results at each testing conditions.

Figure 9 sums up healing test results obtained for each investigated binders in terms of healing index according to Equation (5).

As expected, it can be observed that all the HI values obtained for the investigated binders are rather low, but considering that analysis was performed on plain base bitumens this index is able to detect the impact of self-healing on the overall fatigue performance of these materials. Results generally show that in unaged conditions, binders do not seem to show healing properties dependent on the temperature (or stiffness), probably due to the fact that no significant differences in temperature (or stiffness) were between iso-stiffness and iso-thermal conditions, however the bio-binder seems to show an increase in HI at higher temperature and lower stiffness.

On the contrary, in aged conditions it seems that lower temperatures slightly enhance the healing response of softer binders. Contrarily, the excessive stiffness achieved in iso-thermal condition seems to penalize the healing capability of the harder bitumen. This, apparently, not univocal trend could suggest the existence of an optimal self-healing temperature depending on the type of material.

Overall analysis of HI shows that the bio-binder is characterized by a more marked self-healing capability compared to the investigated plain bitumens. In particular, the bio-oil addition enables an enhanced healing potential as compared to the plain bitumen having the same consistency. 

## 5. Conclusions

The present experimental investigation involved rheological measurements aimed at evaluating the fatigue behavior and related self-healing capability of conventional and innovative bitumen. In particular, two plain bitumen having different penetration grade and a bio-binder (obtained by partially replacing one of the plain bitumen with 10% renewable bio-oil) were selected and tested in both unaged and long-term aged conditions. The effects of some variables such as bitumen type, bio-oil addition, and aging on the healing potential of binders were taken into account. All the healing tests in this study were carried out at relatively high strain levels (beyond the LVE limit). The overall analysis of results allowed the following conclusions to be drawn:

- Bio-binder showed an overall fatigue performance similar to that experienced by the plain bitumen having the same consistency (i.e., penetration grade) in both iso-stiffness and iso-thermal testing conditions. However, at the strain level selected, the softer bitumen and bio-binder showed a faster fatigue damage accumulation compared to the harder bitumen, regardless of the aging conditions;
- From the comparison between iso-stiffness and iso-thermal conditions, results seem to show that lower temperature slightly enhance the healing response of bitumen characterized by a lower consistency. However, the excessive stiffness due to lower temperature seem to penalize the healing capability of the harder bitumen;
- The bio-binder is characterized by a more marked self-healing capability compared to the investigated plain bitumens. In particular, the bio-oil addition has improved the healing potential of the plain bitumen having the same consistency, especially in aged conditions.

This experimental study provides a useful contribution in the deepening of fatigue and self-healing phenomena of bituminous binders concurrently taking into account sustainable needs. Overall, these findings indicate that the use of the studied bio-oil as partial replacement of bitumen does not penalize the global fatigue behavior of plain bitumens having similar consistency, but on the contrary it seems to even improve their self-healing capability. Therefore, this research encourages the use of such bio-binders in road pavements in order to obtain significant benefits in terms of performance and durability. 

## Figures and Tables

**Table 1:** Basic characteristics of investigated binders [19].

Sample	Condition	Penetration (0.1 mm)	Softening Point (°C)
B.50/70	unaged	52	49.7
	aged	25	64.1
B.50/70 + A10	unaged	114	43.5
	aged	39	56.7
B.115	unaged	110	40.8
	aged	37	55.8

**Table 2:** Variation of γ_LVE_ for each material at each temperature and percentage variation due to aging.

Material	Condition	30 °C		25 °C		20 °C		15 °C		10 °C	
		γ_LVE_ (%)	Δγ (%)	γ_LVE_ (%)	Δγ (%)	γ_LVE_ (%)	Δγ (%)	γ_LVE_ (%)	Δγ (%)	γ_LVE_ (%)	Δγ (%)
B.50/70	unaged	2.7	15	2.5	20	2.2	23	1.6	0	1.4	−7
	aged	2.3		2.0		1.7		1.6		1.5	
B.50/70 + A10	unaged	5.7	49	4.3	44	3.0	33	2.0	10	1.8	10
	aged	2.9		2.4		2.0		1.8		1.6	
B.115	unaged	5.7	49	3.9	38	3.1	32	2.4	29	1.8	10
	aged	2.9		2.4		2.1		1.7		1.6	

**Table 3:** Temperatures guarantying the iso-stiffness condition.

|G*|∙sen*δ* (MPa)	T_isostiffness_ (°C)					
	B.50/70		B.50/70 + A10		B.115	
	Unaged	Aged	Unaged	Aged	Unaged	Aged
1.0	36.7	46.3	29.1	39.2	28.0	38.4
3.0	28.3	35.7	22.0	29.5	20.9	29.0
6.0	23.0	29.0	17.6	23.3	16.4	23.0
9.0	19.8	25.1	14.9	19.7	13.8	19.6
12.0	17.6	22.3	13.1	17.2	11.9	17.1

**Table 4:** Testing parameters for healing analysis.

Material	Test Conditions	Unaged			Aged		
		γ_heal_ (%)	T (°C)	|G*|∙sinδ (MPa)	γ_heal_ (%)	T (°C)	|*G*|*sinδ (MPa)
B.50/70	Iso-stiffness	3	28	3	3	35	3
	Iso-thermal	3	25	4.5	2.5	25	10.4
B.50/70 + A10	Iso-stiffness	4	22.3	3	3.5	29.6	3
	Iso-thermal	5	25	1.7	3	25	5.4
B.115	isostiffness	4	22.5	3	3.5	28.6	3
	isothermal	5	25	1.6	3	25	5.2

**Table 5:** Healing parameters in iso-stiffness conditions ($\left|G^{*}\right|\cdot \sin\delta$ = 3 MPa).

Materials	Condition	γ_heal_ (%)	T (°C)	N_0_	N_fH_	n_95_	ΔN_∞_	N_fat_
B.50/70	unaged	3.0	27.7	14,950	282	1.43	1355	15,232
	aged	3.0	35.0	34,350	2182	3.74	980	36,532
B.50/70 + A10	unaged	4.0	22.5	7480	190	1.65	715	7670
	aged	3.5	29.6	19,380	1424	3.20	640	20,804
B.115	unaged	4.0	21.7	7190	202	1.59	720	7392
	aged	3.5	28.6	21,070	705	2.43	650	21,775

**Table 6:** Healing parameters in iso-thermal conditions (T = 25 °C).

Materials	Condition	γ_heal_ (%)	|G*|∙sin*δ*	N_0_	N_fH_	n_95_	ΔN_∞_	N_fat_
B.50/70	unaged	3.0	5.4	14,320	280	0.36	620	14,600
	aged	2.5	10.4	62,640	2802	4.86	560	65,442
B.50/70 + A10	unaged	5.0	2.0	3970	188	0.74	810	4158
	aged	3.0	5.4	24,210	1891	3.61	420	26,101
B.115	unaged	5.0	1.9	4350	120.5	1.46	880	4471
	aged	3.0	4.9	34,030	1509	2.71	630	35,539
